# Reliable water quality prediction and parametric analysis using explainable AI models

**DOI:** 10.1038/s41598-024-56775-y

**Published:** 2024-03-29

**Authors:** M. K. Nallakaruppan, E. Gangadevi, M. Lawanya Shri, Balamurugan Balusamy, Sweta Bhattacharya, Shitharth Selvarajan

**Affiliations:** 1 School of Computer Science Engineering and Information Systems, Vellore Institute of Technology, Vellore, 632014 India; 2 Department of Computer Science, Loyola College, Chennai, Tamil Nadu 600034 India; 3 Shiv Nadar University, Delhi-NCR, 201314 India; 4 School of Built Environment, Engineering and Computing, Leeds Beckett University, Leeds, LS13HE UK; 5 Department of Computer Science, Kebri Dehar University, Kebri Dehar, Ethiopia

**Keywords:** Water Quality, Potability, TDS, Explainable AI, Water Quality Prediction, Engineering, Electrical and electronic engineering

## Abstract

The consumption of water constitutes the physical health of most of the living species and hence management of its purity and quality is extremely essential as contaminated water has to potential to create adverse health and environmental consequences. This creates the dire necessity to measure, control and monitor the quality of water. The primary contaminant present in water is Total Dissolved Solids (TDS), which is hard to filter out. There are various substances apart from mere solids such as potassium, sodium, chlorides, lead, nitrate, cadmium, arsenic and other pollutants. The proposed work aims to provide the automation of water quality estimation through Artificial Intelligence and uses Explainable Artificial Intelligence (XAI) for the explanation of the most significant parameters contributing towards the potability of water and the estimation of the impurities. XAI has the transparency and justifiability as a white-box model since the Machine Learning (ML) model is black-box and unable to describe the reasoning behind the ML classification. The proposed work uses various ML models such as Logistic Regression, Support Vector Machine (SVM), Gaussian Naive Bayes, Decision Tree (DT) and Random Forest (RF) to classify whether the water is drinkable. The various representations of XAI such as force plot, test patch, summary plot, dependency plot and decision plot generated in SHAPELY explainer explain the significant features, prediction score, feature importance and justification behind the water quality estimation. The RF classifier is selected for the explanation and yields optimum Accuracy and F1-Score of 0.9999, with Precision and Re-call of 0.9997 and 0.998 respectively. Thus, the work is an exploratory analysis of the estimation and management of water quality with indicators associated with their significance. This work is an emerging research at present with a vision of addressing the water quality for the future as well.

## Introduction

The major part of our earth comprises water and it is extremely important for the survival of all humans and animal species. Water makes up over 326 cubic metres of the planet’s surface, which is almost 71% of its total area out of which 97% is seawater. Only 0.5 percentage of the drinkable water on earth is accessible, while the remaining 2.5 percentage is either trapped in glaciers, polar ice caps, the atmosphere, on soil, is polluted, or lies beneath the earth’s surface far beyond human reach. If the global water supply is 100 L, consequently the amount of drinking water would be only 0.003 L, which is just a teaspoon. Therefore, the management and preservation of drinking water is regarded as a top priority. It is the most critical issue for mankind to address given the extremely limited amount of water that is accessible for use. The quantum of water around the world is represented in Table 1.

**Table 1:** Water availability around the globe.

Location	Quantity (%)
Oceans	97.2
Ice Caps/Glaciers	2.0
Groundwater	0.62
Freshwater Lakes	0.009
Inland seas/salt lakes	0.008
Atmosphere	0.001
Rivers	0.0001

Water is a common and crucial resource shared among all humans, animals, and plants and is a necessity for all species. Each one of these species has its own respective needs for water quality. Total Dissolvable Solids (TDS) of soft water for human consumption range from the best quality stated, which is between 50 mg/dL and 150 mg/dL. Between 150 mg/dL and 300 mg/dL is the next level that can be applied to humans. The plants need water that is between 700mg/dL and 800mg/dL. The animals, especially cattle consume water around the quality of 1000 mg/dL. It is thus evident from all these observations that water quality management is essential to ensure sustainability and a healthy life on Earth. The impact of water quality prediction is crucial at a global level for many reasons. First of all, to get clean and safe water is a basic human necessity and water quality prediction aids to guarantee the availability of potable water for societies worldwide. Water quality is related to public health as polluted water may cause waterborne diseases which could affect millions of humans globally. A sustainable environment is an important aspect of human well-being by preserving ecosystems and biodiversity. The significance of water quality assessment is profound and intricate by various organizations globally. The WHO (World Health Organization) , UNEP (United Nations Environment Programme), EPA (United States Environmental Protection Agency), EEA (European Environment Agency), IWA (International Water Association) and WEF (Water Environment Federation) are fanatical for water quality assessment and addressing the mitigation strategies for water quality challenges. Water quality creates impact on public health globally and resulting in dissemination of waterborne diseases like typhoid, dysentery, cholera, dengue and malaria and cause substantial risks worldwide.

The advancement in computing technologies and artificial intelligence have elevated the standards of water quality assessments^1^. Measurements and estimations about the quality of the water have become easier to calculate and accurate, especially with the development of Industry 4.0 standards and Internet of Things (IoT) sensors. With the integration of IoT sensors, AI solely serves as a supporting tool to automate water quality checks^2^. Classification and Regression models based on machine learning help in determining the water quality. Depending on the outcomes, classification results tend to be binary or multi-classified. Real-time sensor data are collected, given feature labels, and then classified based on the importance of the feature labels. Earlier, these measurements used to be carried out with fuzzy-based decision support systems^3^ with subjective decision-making models. AI development has made it possible to classify and analyse quality aspects quantitatively. The accuracy of the water quality assessment has been validated using various performance metrics like accuracy, precision, recall, and f1-score. AI models also support such quantitative analysis, classification of water sources, and prediction of drinkable water as well as identifying the mixing of bouyant pollutants in water sources^4^.

Despite its success in automating tasks and making water quality predictions using diverse models, the AI models lack transparency and are considered black-box where the decisions are derived but the reasoning behind such decisions is not revealed. The present generation validation frameworks for water quality management need justifiability, transparency and explainability, which is possible to be rendered by Explainable AI (XAI) based systems. XAI is a technology that is white-box and answers the uncertainty related to the classification and regression problems of AI. XAI applies a model-agnostic approach, where the machine learning models can be treated independently for interpretation. Additionally, XAI discusses how the model is chosen, how it works, and how it performs categorization. Through the assessment of a problem’s feature weights, XAI also can determine a feature’s relevance. This clarifies how a feature value relates to a certain target class classification. As an example, XAI uses models like Partial Dependency Plots (PDP)^5^, which describes the relationship between the features using lasso functions. This model may identify the linear relationship between two characteristics of water quality data and explain their correlation. In XAI, models like Local Interpretable Model agnostic Explainer(LIME), explain the relationship between a single feature and relevant others in local surrogacy. This infers that, except for the one-row value of the dataset, it is possible to relate a target attribute to the other independent variables. LIME in this regard can be used to explain the target classification for a single row instance about the water quality^6^. In the proposed work, XAI, which employs both local and global surrogates, includes SHAPELY. The model offers a solution that takes into account the importance of each feature in determining the target as well as the dependency between features, the relationship between features, and the explanation of decisions through a variety of plots, including force plots, summary plots, dependency plots, and decision plots. The framework is very adaptable and capable of giving a thorough explanation of the characteristics of the water quality and how they affect the classification of the water quality.

### Advantages of the proposed model

Explainable AI plays an important role in improving the interpretability of predictions made by machine learning models. More transparent predictions are generated by these models. In the proposed approach, the authors have employed LIME and SHAP to interpret predictions achieved from machine learning, which identifies inputs as an important metric for selecting the features. By applying the XAI approach, the proposed model provides deep insights into the features and allows informed decision-making in water management processes.

### Contributions of the paper

The following points describe the contribution of the proposed work.

- The proposed work offers a comprehensive analysis and white-box description of the classification problem for water quality.
- The framework incorporates extensive pre-processing of the dataset to ensure it fit to be fed into the XAI model.
- Imputation of missing data is carried out to increase the accuracy of the findings.
- The proposed work ensures achievement of most significant features, identification of the feature importance, feature dependencies, and feature weights, that enable optimized classification of water quality dataset.
- The proposed approach employs both model-based and model-agnostic interpretations, using model-based ML implementations and model-agnostic XAI implementations.

### Organization of the paper

Section “Introduction” of the paper introduces the problem of the research paper with the description of the unique contributions. Section. Introduction” also describes the literature review of the related problems on water quality, in related works subsection, with an exhaustive survey of the various applications and case studies pertaining to water quality management using AI and machine learning approaches. Section “System model and architecture” describes the methods applied in the proposed work with the implementation of the mathematical model with the algorithm of the proposed work. Section “Results” describes the results of various ML and XAI models with relevant tables and graphs. Section “Discussion” provides the comparative analysis of the results with a discussion of challenges and solutions of the proposed work. Section “Conclusion” concludes the paper with future directions.

### Related works

Lu et al.^7^ proposed the central environmental protection inspection (CEPI), which was implemented and the causes of transboundary water contamination were investigated. The triple difference technique (DDD) was used to assess how the CEPI affected pollution and the results to determine how significantly water pollution was decreased as well as the significance of CEPI laws for addressing transboundary pollution. Halder et al.^8^, the Turag River’s neighbouring communities are suffering from major health problems as a result of water contamination. For the sustainability of household and aquatic life, the river’s water quality was unsuitable. The study noted that the threshold values for turbidity, total dissolved solids (TDS), chloride (CL-), chemical oxygen demand (COD), carbon dioxide (CO2), and biochemical oxygen demand (BOD) are higher than the standard permissible limits, which may result in health problems like respiratory illnesses, diarrhoea, cholera, dengue, malaria, anaemia, and skin problems. A study evaluating metal pollution management and mitigation tactics on soil and water was presented by Wang et al.^9^. In this study, the remediation of metal contamination from water and soil utilising chemical, physical, and biological approaches was discussed. In this study, the current methods for reducing heavy metal pollution of the soil and water are examined. Elehinafe et al.^10^ discussed the importance of water contamination and examined the main cause of water scarcity. The proposed work discussed the effect of hazardous chemicals on the water, including pesticides, heavy metals, and micro-pollutants. This study outlined the numerous technologies that are currently available to eliminate hazardous materials and provide sustainable clean water resources. Mu et al.^11^proposed a solution for the investigation into farmers’ readiness to implement Rural Water Pollution Control (RWPC). This study examines farmers’ viewpoints to improve the quality of life for locals who reside in rural regions and avoid water contamination. To analyse the contributions of contaminants, Wang et al.^12^ developed a unique contaminant flux variable model for river water quality assessment. The framework effectively identified the sources of pollution and evaluated the efficacy of projects designed to reduce water pollution. Zadeh et al.^13^ proposed WQPs for estimating chemical oxygen demand and biochemical oxygen demand using the MKSVR algorithm. PSO algorithm is used for solving optimization problems. The multiple kernel support vector regression (MKSVR) is compared with SVR and Random Forest Regression and achieves a better accuracy level for BOD prediction. Nagaf et al.^14^ presented a framework for assessing the WQI values based on the NSF guidelines. This framework uses four data-driven models such as EPR, M5 MT, GEP and MARS for predicting WQI values in the Karun River. The classification uses 12 water quality parameters and missing values were extracted from the image analysis. Zadeh et al.^15^ proposed a model that utilizes gene expression programming, evolutionary polynomial regression, and model trees for predicting WQPs. The biochemical oxygen demand, dissolved oxygen and chemical oxygen demand are used for estimation with nine parameters. The gamma test is used for determining important parameters. Najaf et al.^16^ proposed a water quality predicting framework for estimating the water quality index in the Hudson River based on Canadian Council of Ministers of the Environment (CCME) guidelines. The four artificial intelligence techniques M5 MT, Multivariate Adaptive Regression Spline, Evolutionary Polynomial Regression and Gene Expression Programming are used with Landsat 8 OLI-TIRS images. The results proved that the MARS technique achieved the best outcome compared to other models.

Chowdhury et al.^17^ emphasized the sources of water contamination which are caused by densely populated industrial areas that are located close to water bodies. The main causes of water contamination are dangerous chemicals and heavy metals. Farmers’ pre-owned pesticides, including different types of carbamate and organophosphorus pesticides, are the main causes of water contamination on agricultural grounds as per the study. Ahivar et al.^18^ examined the use of heavy metal pollution indices (HPIs) in soil, water, and sediments. For assessing metal contamination, HPI is considered a crucial instrument. Each method’s pollution index is assessed to interpret the pollution levels. The selection of HPIs based on the parameters and standards for evaluating the quality of the water and soil is offered. Chen et al.^19^ presented a study by used various mathematical and statistical approaches to check the quality of water. The factors indicating the water pollution and the seasonal characteristics are evaluated to reduce the river water pollution. The Principal Component Analysis, Cluster Analysis, Network Analysis and Co-Occurrence Analysis were carried out to find the potential source of river water pollution. Fan et al.^20^ examined the quality of water using several mathematical and statistical techniques. To lessen river water pollution, the variables implicating contamination and the seasonal traits are assessed. To identify a likely cause of river water pollution, the Principal Component Analysis, Cluster Analysis, Network Analysis, and Co-Occurrence Analysis were performed. Wang et al.^21^ formulated the performance indices for explaining the Water-Energy-Pollution nexus (InWEP) effects of scales. The Nexus Pressure Index (NPI) and Nexus Coupling Index (NCI) were used to represent the pollution pressure and the interacted relations. The factors for InWEP were analysed using the Structural Equation Model (SEM) considering four objects namely enterprises, countries, industrial zones and cities. The performance of InWEP was evaluated for the performance metrics - efficiency, structure and location. To evaluate the quality of groundwater surrounding nearby areas in an industrial metropolis, Asomaku^22^ evaluated the water pollution indices. Nine samples from three landfills are used in the analysis of the groundwater’s chemical and metal characteristics. The study in Balaram et al.^23^ explored many elements that have an impact on water quality, including climate change, industry, aquaculture, mining, and agriculture. For the quantitative and qualitative evaluation of hazardous metals, metal species, isotopes, and other contaminants that are present in water, various ICP-MS techniques are applied. Yuan et al.^24^ proposed a water quality monitoring framework using biological sensors for water quality assessment. Borzooei et al.^25^ presented a study to estimate the frequency weather events that creates impact on waste water assessment. The Time series data mining approach is used for categorizing the dry and wet weather events. Noori et al.^26^ presented a report on decline of groundwater recharge in Iran. The study presents the average amount of ground water recharge is more than the annual runoff^4^ utilized WCSPH (A weakly compressible smoothed particle hydrodynamics) model for simulating the near-shore hydrodynamics. The study conducted experimental and numerical evaluation for detecting the causes for mixing the buoyant pollutants in coastal water source. Yeganeh-Bakhtiar^27^ presented a framework using MOS (Model Output Statistics) for establishing the statistical relationships among predicator and predicant.

**Figure 1:**
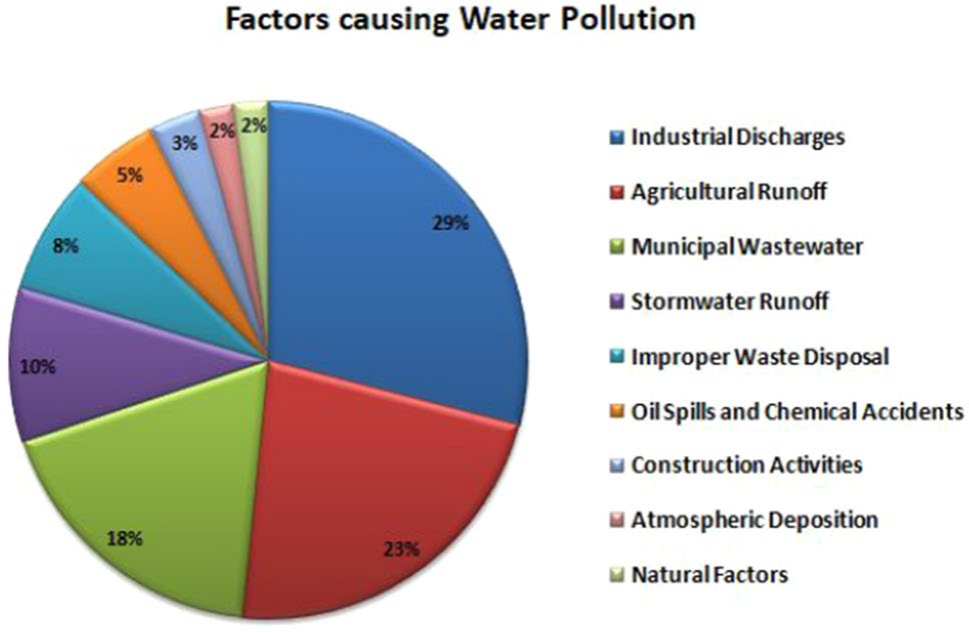
Factors causing water pollution.

When evaluating water quality using factors like toxicity and pollutants, computer vision and biological sensor systems are utilised in tandem. To retrieve the important data from images taken by a microscope, a microfluidic chip with sensors is utilised. This chip monitors water samples. Figure 1 describes various factors causing water pollution in smart cities including construction activities, atmospheric deposition, natural factors, municipal wastewater, stormwater runoff, incorrect waste disposal, industrial discharges, agricultural runoff, and municipal wastewater. Jeihouni et al.^28^ implemented and compared five data mining techniques, including the Ordinary Decision Tree (ODT), Random Forest (RF), Chi-square Automatic Interaction Detector (CHAID), Iterative Dichotomiser 3 (ID3), and Random tree, to identify high-quality water zones. Eight parameters are used in the evaluation process while deriving rules. Compared to the remaining models, the RF performed well, with an accuracy rate of 97.10%. Lee et al.^29^ implemented a framework for evaluating the quality of groundwater utilising a Self-Organizing Map (SOM) technique and fuzzy c-means clustering (FCM) was given. The two methods are employed to describe the complex nature of groundwater. SOM employed 91 neurons to categorise 343 groundwater samples, and FCM grouped the water sources into three groups. Agarwal et al.^30^ proposed AI based water evaluating technique to predict the water quality index using Particle Swarm Optimization (PSO), Naïve Bayes Classifier (NBC), and Support vector machine (SVM). PSO was used in this regard for optimizing the classifiers wherein the PSO-optimized NBC obtained 92.8% accuracy and PSO-optimized SVM obtained 77.60% accuracy. Table 3 illustrates various existing state-of-art techniques proposed for assessing water quality, its advantages and research gaps.

Figure 1 illustrates the factors causing water pollution. The factors includes Industrial discharges, agricultural runoff, municipal waste water, storm water, improper waste disposal, oil spills and chemical spills, construction wastages, and atmospheric deposition. The factors are very crucial to protect public health and ecosystem , sustainability development, creating public awareness and for pollution prevention.

**Figure 2:**
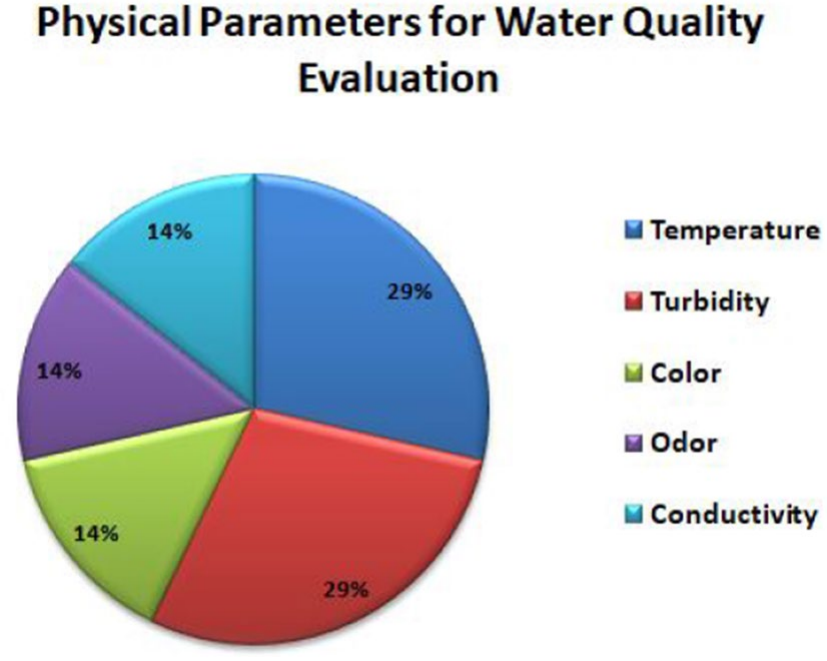
Physical Parameters.

Figure 2 depicts the required physical parameters such as Temperature, Turbidity, Conductivity, Odour and Color represented in percentage, for evaluating the quality of water. Examining the physical parameters is essential for identifying the potential hazards that leads to poor water quality and for preventing ecosystem health.

**Figure 3:**
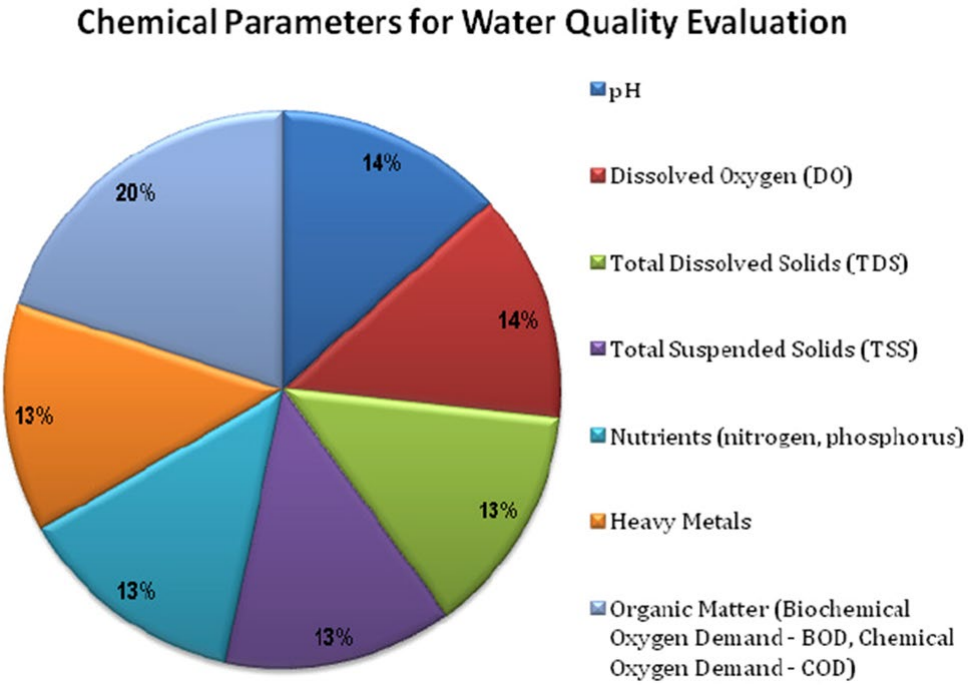
Chemical parameters.

Figure 3 depicts the necessary chemical parameters, such as pH, Dissolved Oxygen (DO), Total Dissolved Solids (TDS), Nutrients (nitrogen and phosphorus), Total Suspended Solids (TSS), Heavy Metals, and Organic Matter (OM), as well as Chemical Oxygen Demand (COD) and Biochemical Oxygen Demand (BOD) with percentages, that must be measured in order to assess the water’s quality.

**Figure 4:**
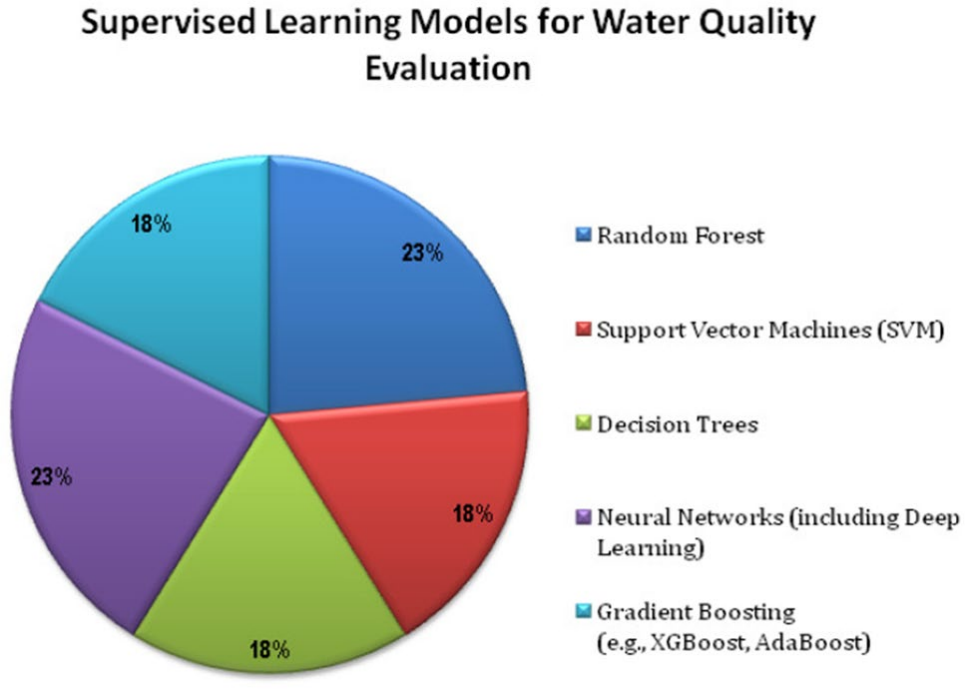
Supervised learning models.

Figure 4 presents various supervised learning models for estimating water quality, including Random Forest, Support Vector Machine (SVM), Decision Trees, Neural Networks, and Gradient Boosting Approaches like XGBoost and AdaBoost.

**Figure 5:**
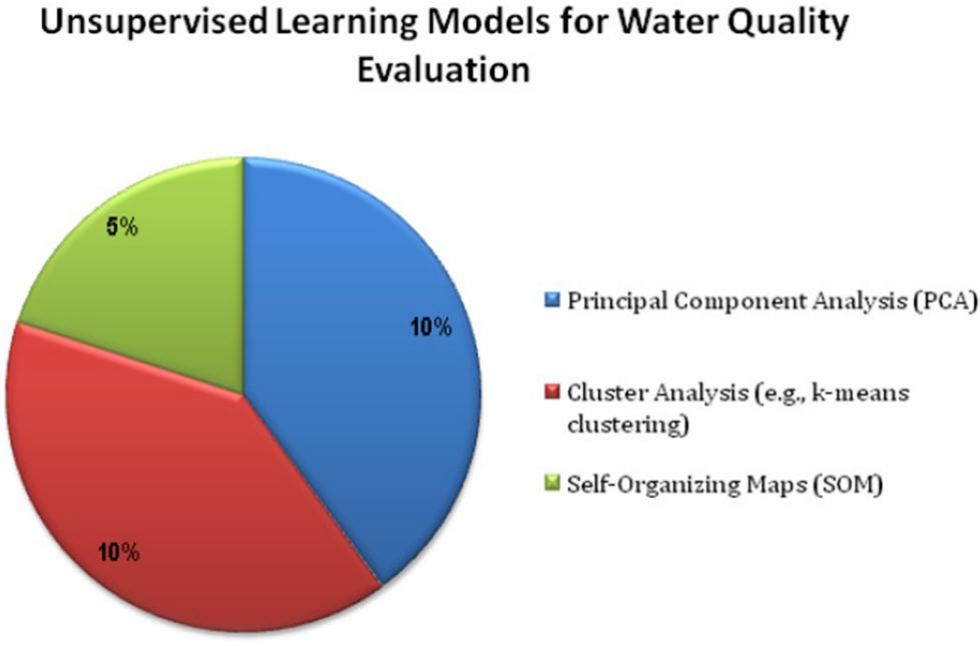
Unsupervised learning models.

Figure 5 represents various unsupervised learning models such as Principal Component Analysis, Cluster Analysis and Self-Organizing Maps (SOM) for addressing the quality of the water. PCA is a dimensionality reduction approach mainly utilized for analyzing the high dimensional datasets. Cluster analysis techniques are used primarily for grouping water samples based on similarities. SOM technique is principally used for organizing the water quality data.

**Figure 6:**
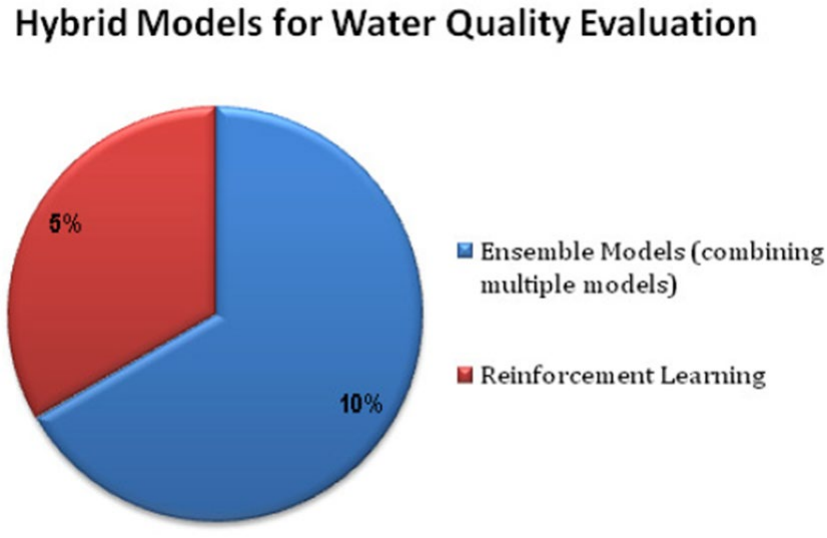
Hybrid ML models.

Figure 6 highlights the various Hybrid ML models such as ensemble models with Reinforcement Learning (RL) for addressing the evaluation of quality of water. The various machine learning models can be verified based on the applications, parameters in order to determine the quality of the water, dataset size and its quality based on the assessment of the performance metrics.

The motivation for the proposed research, along with the research gap analysis with similar existing research works is discussed as per Table 2. The comparative analysis and research of similar existing works are presented in Table 3. These two discussions provide a comprehensive understanding of the requirements, that are essentially required in the design of the proposed system and implementation.

**Table 2:** Motivation for the proposed work from the review perspective.

Refs	Title	Advantages	Research gap	Quality parameters
^24^	A biological sensor system using computer vision for water quality monitoring	$\bullet$ Real-time analysis	$\bullet$ Sensor Sensitivity and Selectivity	$\bullet$ Abstract Fish Behavior
		$\bullet$ Cost Effectiveness	$\bullet$ Robustness and Long term stability	$\bullet$ Movement Velocity
		$\bullet$ Versatility	$\bullet$ Calibration	$\bullet$ Rotation Angle Of The Fish Group
		$\bullet$ Potential for Automation	$\bullet$ Image Analysis and Algorithm Optimization	
^31^	Monthly water quality forecasting and uncertainty assessment via bootstrapped wavelet neural networks under missing data for Harbin, China	$\bullet$ Accurate Water quality forecasting	$\bullet$ Model Generalization	$\bullet$ Ammonia Nitrogen (NH4+-N)
		$\bullet$ Handling Missing data	$\bullet$ Comparison with other Models	$\bullet$ Dissolved Oxygen
		$\bullet$ Uncertainty Assessment	$\bullet$ Data Availability and Quality	
		$\bullet$ Temporal Resolution	$\bullet$ Model Optimization	
^32^	Groundwater quality forecasting using ML algorithms for irrigation purposes	$\bullet$ Spatial distribution mapping	$\bullet$ Spatial and temporal scale	$\bullet$ Total Dissolved Solid (TDS)
		$\bullet$ Effective feature selection	$\bullet$ Data availability and quality	$\bullet$ Potential Salinity (PS)
		$\bullet$ High dimensional data	$\bullet$ Uncertainty estimation	$\bullet$ Sodium Adsorption Ratio (SAR)
		$\bullet$ Non-linearity and flexibility	$\bullet$ Validation and comparison	$\bullet$ Exchangeable Sodium Percentage
			$\bullet$ Transparency	$\bullet$ Magnesium Adsorption Ratio (MAR)
				$\bullet$ Residual Sodium Carbonate (RSC)
^33^	Predicting nitrate concentration and its spatial distribution in groundwater resources using Support Vector Machines (SVM) model	$\bullet$ Accurate Water quality forecasting	$\bullet$ Model Generalization	$\bullet$ Water Temperature
		$\bullet$ Handling Missing data	$\bullet$ Comparison with other Models	$\bullet$ Electrical Conductivity
		$\bullet$ Uncertainty Assessment	$\bullet$ Data Availability and Quality	$\bullet$ Groundwater Depth
		$\bullet$ Temporal Resolution	$\bullet$ Model Optimization	$\bullet$ Total Dissolved Solids
		$\bullet$ Site-Specific Application		$\bullet$ Dissolved Oxygen
				$\bullet$ Ph
^34^	A novel ML-based approach for the risk assessment of nitrate groundwater contamination	$\bullet$ Risk assessment accuracy	$\bullet$ Dataset limitations	Groundwater Vulnerability Map :(DI<80), low (DI=80-120), moderate (DI=120-160), high (DI=160-200), and very high
		$\bullet$ Ability to handle complex datasets	$\bullet$ Can exhibit temporal dynamics	
		$\bullet$ Spatially explicit risk mapping	$\bullet$ Validation	
		$\bullet$ Transferability to different regions	$\bullet$ Uncertainty quantification	
				$\bullet$ Comparative analysis
^35^	Machine Learning predictions of nitrate in groundwater used for drinking supply in the conterminous of the United States	$\bullet$ Nationwide assessment	$\bullet$ Data quality and availability	$\bullet$ High Precipitation
		$\bullet$ Accuracy and predictive power	$\bullet$ Incorporating temporal dynamics	$\bullet$ Recharge
		$\bullet$ Detection of the high risk areas	$\bullet$ Transferability and regional variability	$\bullet$ Base Flow Index
		$\bullet$ Spatially explicit predictions	$\bullet$ Uncertainty estimation	$\bullet$ Nitrate Concentrations
			$\bullet$ Comparative analysis	
^36^	Ensemble modelling framework for groundwater level prediction in urban areas of India	$\bullet$ Model training and calibration	$\bullet$ Data quality and availability	$\bullet$ Groundwater Levels
		$\bullet$ Ensemble generation	$\bullet$ Transferability and regional variability	$\bullet$ Rainfall, Temperature
		$\bullet$ Uncertainty estimation	$\bullet$ Validation benchmarking	$\bullet$ NOI
		$\bullet$ Enhanced pre-processing techniques	$\bullet$ Comparative analysis	$\bullet$ SOI
				$\bullet$ NIÑ
				$\bullet$ Monthly Population Growth Rate

**Table 3:** Comparative Analysis from the review perspective.

Reference	Algorithms	Input parameters	Evaluation results
^37^	PNN	BOD, PO4-P, COD, temperature, NO 3-N, Ca 2+, Cl-, alkalinity, P, Mg 2+, pH,and EC	Interpolation is good performance - R2 : 0.82
^31^	BWNN, ANN, ARIMA, BANN	Dissolved Oxygen	ARIMA < ANN<WNN <BANN<BWNN
^38^	LSTM	Dissolved Oxygen	High runoff ratio $\ge$ 0.45 $\bullet$ - 74% of sites
^39^	CCNN	water quality and DO parameters (example: Cl, NO x,pH, TDS, and temperature)	R2 : 0.825 RMSE: 0.550
^40^	SVM, ANN	TDS, Na+, Mg 2+,Temperature, pH, EC, HCO3 , Cl, and Ca 2+	SVM performs better than ANN
^41^	SVM, ANN	flow travel time, rainfall, river flow, temperature, DO, TN, and TP	SVM perform better than ANN
^42^	DT, RF, DCF, and 10 other models	pH, DO, CONMn, and NH 3-N	DCF, DT, and RF performed well
^43^	SVR	EC, fDOM, turbidity, BGA-PC, chlorophyll-a, DO, and sediments	BGA-PC : (Accuracy: 0.77), chlorophyll-a (Accuracy: 0.78), TSS (Accuracy: 0.81), from (-), turbidity (Accuracy: 0.55)
^44^	Attention-based neural network	Images of water	Accuracy: Polluted-water= 73.6% Accuracy: Clean-water=71.2%
^45^	SVM, RF, CNN	Landsat8 images	RF Accuracy: 86.21% SVM Accuracy: 96.89% CNN Accuracy: 97.12%

Table 3 refers to similar literature review of various models of machine learning such as DT,RF,DCF, SVM, and so on. This table also discusses about various deep learning models such as, Artificial Neural Networks (ANN), Probablistic Neural Network (PNN), Convolution Neural Networks (CNN) and statistical regression models such as Auto-Regression in Moving Average(ARIMA). This table discusses the the research gaps identified and enhanced in the proposed work. These models were mostly numerical evaluations with regression analysis. The proposed model and the system is classifier which deploys XAI framework, to discuss the impact of parameters, that determine the portability of the water with end user perspective. This is towards achieving environmental sustainability on water conservation and harvesting.

### Statement of objectives

The proposed work offers a comprehensive analysis and white-box description of the classification problem for water quality . The framework incorporates extensive pre-processing of the dataset to ensure it fits into the XAI model. Imputation of missing data is carried out to increase the accuracy of the findings. The proposed work ensures the achievement of the most significant features, identification of the feature importance, feature dependencies, and feature weights, that enable optimized classification of the water quality dataset. The proposed approach employs both model-based and model-agnostic interpretations, using model-based ML. Donnelly et al.^46^ implementations and model-agnostic XAI implementations. The quality of water is greatly challenged by innumerable influencing factors. These factors vary from condition to condition and place to place. For example, Microplastics (MP) are emerging pollutants in the marine environment with potential toxic effects on littoral and coastal ecosystems^47^ and as well as identifying the mixing of bouyant pollutants in water sources^4^. The laboratory evaluations show the presence of polyethene (PE) particles in the waves of the ocean with wave steepness Sop of 2–5%. The transportation of which could cause severe water pollution on the seashores^48^.These measurements require quantification and feature analysis when it is evaluated with AI. This is where the XAI plays a vital role in measuring the order and degree of the pollutants causing the quantifiable pollution in the water.

### Case studies

Importance of XAI in Water Quality Assessment: The following case studies delineate the advent of the potential impact of XAI, with a groundbreaking revolution in water quality assessment.

Case Study 1: Pollution of Ganges^49^ This case study emphasises the Ganga River pollution issue in India, which has an extremely detrimental impact on humans and the entire ecosystem. The Ganga River is polluted by industrial, animal, and human waste. The main source of pollutants is industrial rubber waste, followed by leather and plastic manufacturers who dump their untreated wastewater into the river. The Ganga Action Plan was developed by the Indian government to combat Ganga pollution. This implies the need for the reinforcement of environmental restrictions to improve river quality.

### Materials and methods

An effective policy for health protection should thus emphasize providing access to safe drinking water regardless of social and economic diversity. In some places, it is evident from previous studies that investments in access to clean water and sanitation yield economic benefits for any country. It is a significant aspect of eco-friendly health and public safety, as it regulates the appropriateness of water for numerous purposes, such as drinking, agriculture, industry, and recreational purposes. The important key indicators related to water quality are its physical, chemical, and biological characteristics and its sources of pollution. The dependent target class is potability. The other independent features are pH value, hardness, solids (Total Dissolved Solids-TDS), Chloramines, sulfate, conductivity, organic carbon, trihalomethanes, and turbidity. Water’s potability indicates its purity and safety for ingestion. The parameters used and their WHO limits, the hyper-parametric analysis are listed in Table 4, and the feature description of parameters are listed in Table 5.

**Table 4:** Hyper parameter analysis of various ML models.

Model	Parameters	Values
Logistic regression	$\bullet$ Penalty	$\bullet$ None
	$\bullet$ Dual	$\bullet$ False
	$\bullet$ Tolerance	$\bullet$ Default
	$\bullet$ Regularization Strength	$\bullet$ Default =1.0
	$\bullet$ Fit_Intercept	$\bullet$ True (Boolean)
	$\bullet$ Class_Weight	$\bullet$ True(Boolean)
	$\bullet$ Random_state	$\bullet$ 0 (Default)
	$\bullet$ Solver	$\bullet$ lbfgs(Default)
SVM	$\bullet$ C	$\bullet$ 1,0
	$\bullet$ Kernal	$\bullet$ Linear
	$\bullet$ Degree	$\bullet$ 3
	$\bullet$ Gamma	$\bullet$ Scale
	$\bullet$ Co_ef	$\bullet$ 0
	$\bullet$ Shrinking	$\bullet$ True(Boolean)
	$\bullet$ Tolerance	$\bullet$ False(Boolean)
	$\bullet$ Cache	$\bullet$ Default
	$\bullet$ Class_weight	$\bullet$ 200MB
	$\bullet$ Verbrose	$\bullet$ None
	$\bullet$ Maximum_Iteration	$\bullet$ False
	$\bullet$ decision_function_shape	$\bullet$ 1
	$\bullet$ break_ties	$\bullet$ ovr(one vs rest)
	$\bullet$ random_state	$\bullet$ False
	$\bullet$ Random_state	$\bullet$ None
Decision Tree	$\bullet$ Criterion	$\bullet$ Gini
	$\bullet$ Splitter	$\bullet$ Best
	$\bullet$ Max_Depth	$\bullet$ None
	$\bullet$ Minimum_samples_split	$\bullet$ 2
	$\bullet$ Minimum_samples_leaf	$\bullet$ 1
	$\bullet$ Minimum_weight_fraction_leaf	$\bullet$ 0
	$\bullet$ Max_features	$\bullet$ None
	$\bullet$ Random_state	$\bullet$ None
	$\bullet$ Minimum_impurity_decrease	$\bullet$ 0
	$\bullet$ Maximum_leaf_nodes	$\bullet$ None
	$\bullet$ Random_state	$\bullet$ None
Random Forest	$\bullet$ N-estimators	$\bullet$ 100
	$\bullet$ Criterion	$\bullet$ Gini
	$\bullet$ Max_Depth	$\bullet$ None
	$\bullet$ Minimum_samples_split	$\bullet$ 2
	$\bullet$ Minimum_samples_leaf	$\bullet$ 1
	$\bullet$ Minimum_weight_fraction_leaf	$\bullet$ 0
	$\bullet$ Max_features	$\bullet$ None
	$\bullet$ Random_state	$\bullet$ 0
	$\bullet$ Minimum_impurity_decrease	$\bullet$ 0
	$\bullet$ Maximum_leaf_nodes	$\bullet$ None
	$\bullet$ BootStrap	$\bullet$ True
	$\bullet$ oob_score	$\bullet$ False
	$\bullet$ n_jobs	$\bullet$ 0
	$\bullet$ Verbrose	$\bullet$ None
	$\bullet$ Class_weight	$\bullet$ 0

XAI framework facilitates transparent and interpretable explanations of the outcome generated by the ML algorithm-based frameworks. XAI can thus be applied in the present context of water quality assessment to ensure accurate decision-making, thereby, enabling trustworthiness, enhancement of transparency and interpretability of the behaviour of the model.

#### Hydro-climatic application

XAI framework can be used to solve Hydro-Climatic problems^50^ with diverse spatio-temporal scales. XAI is utilized to unveil the nonlinear correlative causes, in which the performance of the model is enhanced. It enables the users to discover new knowledge and further easily understand the rationale behind the decision outcomes.

#### Groundwater potential predictions

XAI approach can explain the decisions made by ML models for groundwater potential prediction. The user can easily interpret the outcomes and further comprehend the underlying for an outcome in the realm of water quality evaluation for conservation, and sustainability of water management.

#### Water quality predictions

XAI framework can forecast water quality using metrics and factors with interpretable results. Water quality assessment managers can comprehend the variables and parameters used for outcomes. This forces quality managers to mitigate water quality issues.

#### Flood hazard risk predictions

Floods can trigger landslides from excessive rainfall. Flooding causes countless casualties and property damage. Disaster warning systems need a flood risk assessment. XAI can forecast rapid water depths and provide timely, interpretable alerts to protect public health and safety.

#### Environmental impact assessment

XAI approach can be used for assessing the environmental impact on the water pollution incidents, and provide insight for mitigation and management. It enhances transparency and accountability by providing insights into the factors and parameters influencing environmental conditions. The analysis provided by the XAI model helps the stakeholders to identify the most significant factors contributing towards the environmental outcome.

**Table 5:** Feature description.

Parameters	WHO limits
PH	6.5–8.5
Hardness	200 mgL%
Solids	1000 ppm%
Chloramines	4 ppm%
Sulfate	1000 mgL%
Conductivity	400 μS/cm%
Organic carbon	10ppm%
Trihalomethanes	80 ppm%
Turbidity	5 NTU%

## System model and architecture

### System model

**Figure 7:**
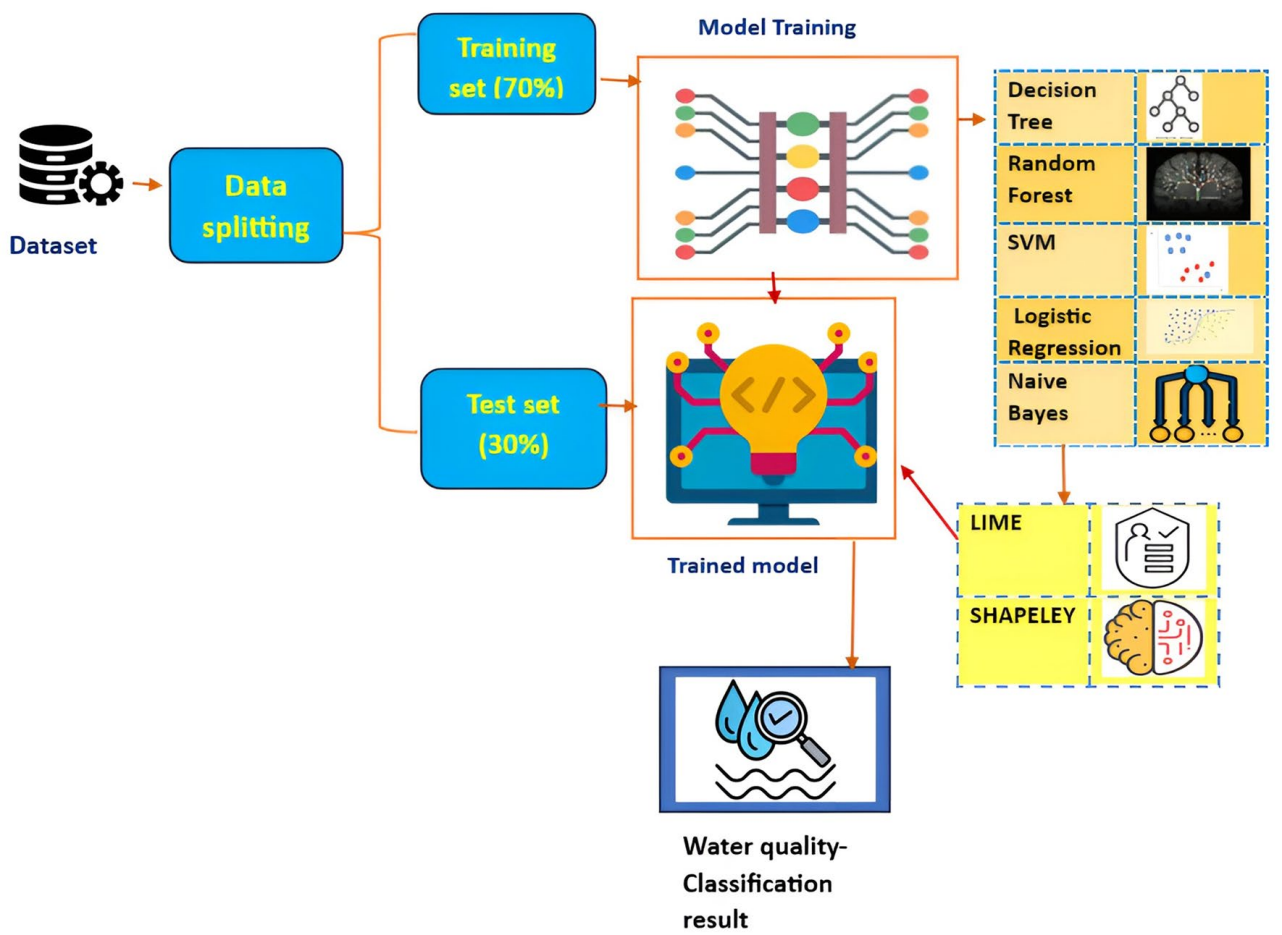
Interfacing ML algorithms with XAI.

Worldwide, numerous water bodies are contaminated by a variety of anthropogenic and natural processes, resulting in a variety of health problems for human life. Thus water quality requires rigorous monitoring and management to prevent pollution. In accordance with WHO guidelines, the polluted water must be treated using the proper water treatment techniques before consumption. The quality of water is contaminated by the incessant addition of toxic chemicals and microbes and also by the relentless addition of local and industrial sewage sludge, trash, and extra hazardous waste that are toxic to humans and society. Many uncertainties are required to be quantified for all machine learning models. The uncertainties such as selecting and gathering the training data, absolute and accurate training data, understanding the machine learning models with performance bounds and drawbacks and finally the uncertainties which are based on the operational data. To minimize the challenges, adhoc steps like studying the model variability and sensitivity analysis are applied. In current years, the validation of water quality has taken active momentum because of ever-increasing water pollutants which spoil water that is dedicated for domestic use and irrigation. Water quality indices (WQIs) are used worldwide very efficiently for the assessment of the quality of both groundwater and other relevant water sources. Machine Learning techniques play a substantial role in identifying the quality of water using explainable AI. Figure 7 depicts the overall architecture of the proposed framework of our study. The dataset used in the study is split into the ratio of 70:30 wherein 70% is used for training and 30% is used for testing. The model is trained using a decision tree, random forest, SVM, logistic regression, and Naive Bayes algorithms. XAI model is implemented in the framework wherein LIME and Shapely are used to provide explainability and interpretability to the results generated by the machine learning model .

#### Decision tree

The decision tree is stated as a recursive partition of the set of all possible instances^27^^51^. The goal of a decision tree is to split the data which consequences in maximum information gain^52^. Let L be a sample for learning, L= ($v_{1}$, $c_{1}$), ($v_{2}$, $c_{2}$),($v_{i}$,$c_{j}$). Here, $v_{1}$, $v_{2}$, $v_{3}$ ,$v_{i}$ are represented for measurement vectors, and $c_{1}$, $c_{2}$, $c_{3}$,$c_{j}$ are represented for class labels.The batch conditions are reliant on one of the vector variables denoted as $s_{i}$^53^. Let us assume if the $e_{i}$ of an element fits class label $c_{i}$, then $p_{i}$ is denoted as per the Eq. (1).

1 $$\begin{aligned} p_{i=}\frac{c_{i}}{L} \end{aligned}$$

Entropy evaluates the random value from the given samples and the homogeneity of the expected rate of a group of data^54^. To divide the data most optimally, the lowest value of entropy signifies better homogeneity.

2 $$\begin{aligned} E\left( L\right)= & {} \left( \frac{c1}{L}\right) {log}_2\left( \frac{c1}{L}\right) +\left( \ \frac{c2}{L}\right) {log}_2\left( \frac{c2}{L}\right) +\ldots +\left( \ \frac{cj}{L}\right) {log}_2\left( \frac{cj}{L}\right) \end{aligned}$$

3 $$\begin{aligned} E\left( L\right)= & {} \ e_{1}{log}_{2}e_{1}+e_{2}{log}_{2 }e_{2 }+\ldots +e_{n}{log}_{2\ }e_{n} \end{aligned}$$

4 $$\begin{aligned} E\left( L\right)= & {} -\sum _{i=1}^{j}{e_{i}{log}_{2 }e_{i}} \end{aligned}$$

L represents the data set evaluated by the entropy, ‘i’ denotes the classes in the set L, and $e_{i}$indicates the number of data labels that fit class ’i’^55^. The least value of entropy is used for choosing the best feature. Information gain enumerates the amount of information provided by a particular characteristic about the target variable to minimize the uncertainty present in the data set. It is calculated by comparing the weighted average of entropy to the original data set after the splitting process. Let us assume that R is the rate for the features ‘f’,$[|{L}^R|]$ denotes the subset of LS so that bf=R^56^. After splitting L on the feature, information gain is given as follows.

5 $$\begin{aligned} IG\left( L,bf\right) =Entropy\ \left( L\right) -\ \sum _{R=1}^{R}\frac{\left| {L}^R\right| }{\left| LS\right| }\ {Entropy\left( {L}^R\right) } \end{aligned}$$

The Gini index evaluates the heterogeneity of a selected node in the decision tree. It counts the probability of wrongly identifying data in the node. The Gini index begins from the value 0 to 1, where 0 indicates a pure node and 1 denotes a node that is distributed equally. The Gini index is represented as

6 $$\begin{aligned} Gini\left( L\right) =1-\ \sum _{i=1}^{j}e_i^2 \end{aligned}$$

Here, $e_{i}$ represents the quantity of data labels. When the data is divided on class d as L1 and L2 with sizes $s_{1}$ and $s_{2}$, Gini is evaluated as

7 $$\begin{aligned} {Gini}_d\left( L\right) =\ \frac{s_1}{s}\ Gini\ \left( {L}_1\right) +\ \frac{s_2}{s}\ Gini\ \left( {L}_2\right) \end{aligned}$$

Due to its comprehensible nature, decision trees can manage both numerical and categorical data with automatic feature selection.

#### Random forest

Random forest is an ensemble method that groups the results of multiple decision trees to compute predictions with enhanced accuracy. Every decision tree is improved on a random subset of labels from the dataset, to achieve diversity between the trees. When the data in the training label is t, then with replacement ‘n’ data are verified as bootstrap data^57^. This is done to produce the decision tree with training data. When there are ’m’ labels, a$<<$m is selected so that ‘a’ values are considered at random from ‘m’. The value ‘a’ is constant when the tree is growing to the highest level. The highest vote is noted as a new instance. (GE*) is the generalization error for the random forest and is denoted as

8 $$\begin{aligned} {GE}^*= P_{x,y}\left( f\left( X,Y\right) \right) <0 \end{aligned}$$

Here, f(X, Y) is a margin function to count the average number of votes from (X, Y). X denotes the prediction value and Y denotes the classification problem. The margin function is represented as

9 $$\begin{aligned} f\left( X,Y\right) ={av}_kF\left( h_k\left( X\right) =Y\right) -{max}_{j\ne y}{av}_kF\left( h_k\left( X\right) =j\right) \end{aligned}$$

where ’F’ is for the indicator function. The value for the margin function is indicated as

10 $$\begin{aligned} R=\ E_{X,Y}\left( f\left( X,Y\right) \right) \end{aligned}$$

The average value of a random forest and the mean correlation of the classifiers are combined as generalization errors. The *p* denotes the mean of the correlation. The generalization error for the upper bound is

11 $$\begin{aligned} {GE}^*\le \rho (1-s^2)/s^2 \end{aligned}$$

Random forest reduces the over-fitting problem compared to a single decision tree. It can effectively manage high-dimensional data.

#### Support vector machine (SVM)

Let us consider a binary classification problem 1 or −1 to represent the sample variables^58^. When i elements of the sample variable is − 1, it is a positive class. When the i variables of the samples is 1, it is a negative set. Let V_i  = X1, X2,...Xn, Yi, i = 1,2,...n, $Y\_{i}\in {-1,1}$, Si indicates i item from the samples. Yi is the i item of the tests performed^59^. To split the samples into two parts, the function f(X) = ZTX+ b is used, where Z is the coefficient vector to normalize the hyperplane. The optimal margin is given as

$$\underbrace{MIN}_{\begin{array}{c} w, b, \\ \varepsilon \end{array}} \left( {\frac{1}{2}}Z^{TZ}+C\sum _{i=1}^{n}\varepsilon _i\right)$$

subject to:

12 $$\begin{aligned} Y_i\left( Z^TX_i+b\right) \ge 1-\varepsilon _i,\ \varepsilon _i\ge 0 \end{aligned}$$

The Lagrangian equation is given as

$$\underbrace{MAX}_{\propto } \left( \sum _{i=1}^{n}{\propto _i-\frac{1}{2}}\sum _{i,j=1}^{n}{\propto _i\propto _jY_iY_jX_iX_j}\right)$$

subject to:

13 $$\begin{aligned} 0\le \propto _i\le C,\ i=1,2,\ldots ,n,\sum _{i=1}^{n}{\propto _iY_i=0} \end{aligned}$$

The Lagrangian equation with the maximum value with $\propto _i$a positive multiplier for the equation $\sum _{i=1}^{n}{\propto _iY_i=0}$ and $\propto _i\ge 0$ to change the optimal hyperplane^60^ is presented. The optimal equation is given as

14 $$\begin{aligned} f\left( X,\propto ^*,\ b^*\right)= & {} \ \sum _{i=1}^{n}{Y_i\propto _i^*<X_i,\ X_j>\ +\ b^*} \end{aligned}$$

15 $$\begin{aligned}= & {} \sum _{i\epsilon sv}^{sv}{Y_i\alpha _i^*<X_i,X_{j\ }>\ +} b^*\end{aligned}$$

In the above equation $\propto _i=0$ of the Lagrangian multiplier is nearest to the margin of the optimal hyperplane denoted as a support vector. This data is linearly separable by the kernel to evaluate the expected result from the instance^61^. The kernel function is denoted as

16 $$\begin{aligned} K\left( X_i,X_j\right) =\ \varphi \left( X_i\right) ^T.\varphi (X_i) \end{aligned}$$

The generalized linear equation is changed to represent the non-linear dual Lagrangian $La(\alpha )$.

$$Lag\left( \propto \right) =\ \sum _{i=1}^{n}{\propto _i-\frac{1}{2}\sum _{i,j=1}^{n}{\propto _i\propto _jY_iY_jK\left( X_i,X_j\right) }}$$

Subject to:

17 $$\begin{aligned} 0\le \propto _i\le C,i=1,2,\ldots n,\sum _{i=1}^{n}{\propto _iY_i=0} \end{aligned}$$

The Lagrangian equation can be used for the separable case as

18 $$\begin{aligned} f\left( X,\ \propto ^*,\ b^(*)\right) =\ \sum _{i=1}^{n}{Y_i\propto _i^(*) K \left( X_i,X_j\right) +\ b^*} \end{aligned}$$

The SVM algorithm is very effective when the quantity of features is higher than the number of samples^62^.

#### Logistic regression

Logistic regression is used for binary classification problems to forecast the probability of an occurrence matching to a particular class. If the dependent value is binary, a regression analysis is used. The idea in logistic regression(logreg) is the logarithm ‘logn’ of odds of X, and odds are the ratios of probabilities ‘pb’ of X^63^. The rate of the independent value is termed odds because logistic regression measures the probability of an act that happens over the likelihood of an occurrence that does not happen.

19 $$\begin{aligned} logreg\left( Y\right) =logn\left( odds\right) =logn\left( \frac{pb}{1-pb}\right) =a+\beta x \end{aligned}$$

where p is the probability of a positive output and x is the variable. The $\alpha$ and $\beta$, are the logistic regression parameters^64^. The above equation is used for finding the number of occurrences as

$$p=probability(Y=positive\ outcome|X=x,$$a specific value)

20 $$\begin{aligned} =\ \frac{e^{\alpha +\ \beta x}}{1+\ e^{\alpha +\ \beta x}}\ \ \ \ \end{aligned}$$

For multiple predictors, a logic regression equation can be written as

21 $$\begin{aligned} logreg\left( X\right) =logn\left( odds\right) =logn\left( \frac{pb}{1-pb}\right) =a+\beta _1x_1+\ldots +\beta _kx_k \end{aligned}$$

$$p=probability(Y=positive\ outcome|X_1=x_1,\ldots ,x_k)$$

22 $$\begin{aligned} =\ \frac{e^{\alpha +\ \beta _{x1}+\ldots +\beta _{xk}}}{1+\ e^{\alpha +\ \ \beta _{x1}+\ldots +\beta _{xk}}} =\frac{1}{1+e^{\alpha +\ \ \beta _{x1}+\ldots +\beta _{xk}}} \end{aligned}$$

Here, pb refers to the probability of the positive occurrence of the event, the Y-intercept is $\alpha$, the regression coefficient is $\beta$, and e is 2.71828. Logistic regression is applied in various domains like finance, healthcare, social sciences, and many more for predicting diseases, credit default, etc.

### Naive Bayesian classification

Gaussian Naive Bayes is a probabilistic classification algorithm developed based on Bayes theorem. It refers to the features which represent a normal distribution^65^. It classifies the samples as most likely classified as

23 $$\begin{aligned} P\left( \left( C_i \mid Y\right) ={\text {Max}}\left\{ P\left( C_1 \mid Y\right) , P\left( C_2 \mid Y\right) , \ldots P\left( C_n \mid Y\right) \right\} \right. \end{aligned}$$

If the sample $Y_{j}$ is a vector, $x_{j}$ is the $j^{th}$ value which contains different values of $y_{j}$. The attributes used are dependent and it is shown as

24 $$\begin{aligned} P\left( Y \mid C_i\right) =\prod _{j=1}^k P\left( A_j=\left( y_j \mid C_i\right) \right) \end{aligned}$$

Substituting the above equation into Bayes classification, we get

25 $$\begin{aligned} P\bigg ((C_i \mid Y)= & {} \frac{\prod _{j=1}^k P(A_j=(y_j \mid C_i) P(C_i)}{P(Y)}\bigg ) \end{aligned}$$

26 $$\begin{aligned} \text { If } \frac{1}{P(Y)}= & {} \propto (>0), \text{ then } \end{aligned}$$

27 $$\begin{aligned} P(C_i \mid Y)= & {} \propto \prod _{j=1}^k P(A_j=(y_j \mid C_i) P(C_i). \end{aligned}$$

The Gaussian Naive Bayes algorithm is mainly applied for spam filtering, sentiment analysis, and text classification problems where the features must be continuous and follow the Gaussian distribution^66^.

#### LIME (Local interpretable model-agnostic explanations)

LIME explains the predictions of any kind of classifier by approximating locally along with an interpretable system. It changes the data sample by altering the values of features and monitors the impact of the result. It explains the predictions from every sample^67^. To receive the labels for the current data, alter the samples *z*’s into the unique form $z \in {\mathbb {R}}^d$. Since the samples *x*’ are generated randomly, x samples closer to the unique instance z for weighing are considered. The weight is evaluated as $\Pi _z(x)$for measuring the intimacy between the data z to x. The currently weighted data *X* and the samples formed by *f*(*x*), are trained as $g \in G$, where *G* is a model. The interpretable model $\xi (x)$ of the current data *g* for explaining *f*(*x*) as

28 $$\begin{aligned} \xi \left( z\right) = {\hspace{0.6cm} g\in G}{\arg {\min }\left( L(f, g,\Pi _{z}) + \Omega (g)\right) } \end{aligned}$$

*L* is the loss function to measure whether *g* is following the state of *f* in the nearest neighborhood of *z*. If the loss function is reduced, the behaviour of *g* takes the behaviour of *f* as $\Pi _z$. The complexity of the model $\Omega (g)$ should be low. When $g(x')$ is considered as a linear function, $g(x') = \varphi ^T x' + \varphi _0$, changes the equation into a linear regression task to evaluate $\varphi$ and $\varphi _0$.

29 $$\begin{aligned} L(f, \varphi _0, \Pi _x) = \sum _{z, z' \in Z} \Pi _x(z) \left( f(z) - (\varphi _0 + \varphi ^T z') \right) ^2 \end{aligned}$$

#### SHAP (SHAPELY Additive exPlanations)

SHAP values determine the status of each feature for the prediction of a specific class^68^. The prediction *f*(*y*), using $s(y')$, a model for the binary elements $x' \in \{0,1\}^M$ with the sets $\emptyset _i \in {\mathbb {R}}$, is given as

30 $$\begin{aligned} s{(x}^\prime )=\ \emptyset _0+\sum _{i=1}^{M}{\emptyset _ix_i^\prime } \end{aligned}$$

*M* refers to the explanation variable.

31 $$\begin{aligned} \Phi _i(f,z) = \sum _{x' \subseteq z'} \frac{(|x'|!(M-|x'|-1)!)}{M!} [f_y(x') - f_y(x'\setminus i)] \end{aligned}$$

where *f* is the model of the SHAP, *z* refers to the variable, and $z'$ are the variables chosen. The value $f_y(x') - f_y(x'\setminus i)$ indicates all the predictions.

### Algorithm

In this section two algorithms are discussed: one for the algorithm-based evaluation of water quality 1 and another for the algorithm-based explanation of water quality 2. These two algorithms provide a holistic analysis and explanation of water quality management.

**Algorithm 1:**
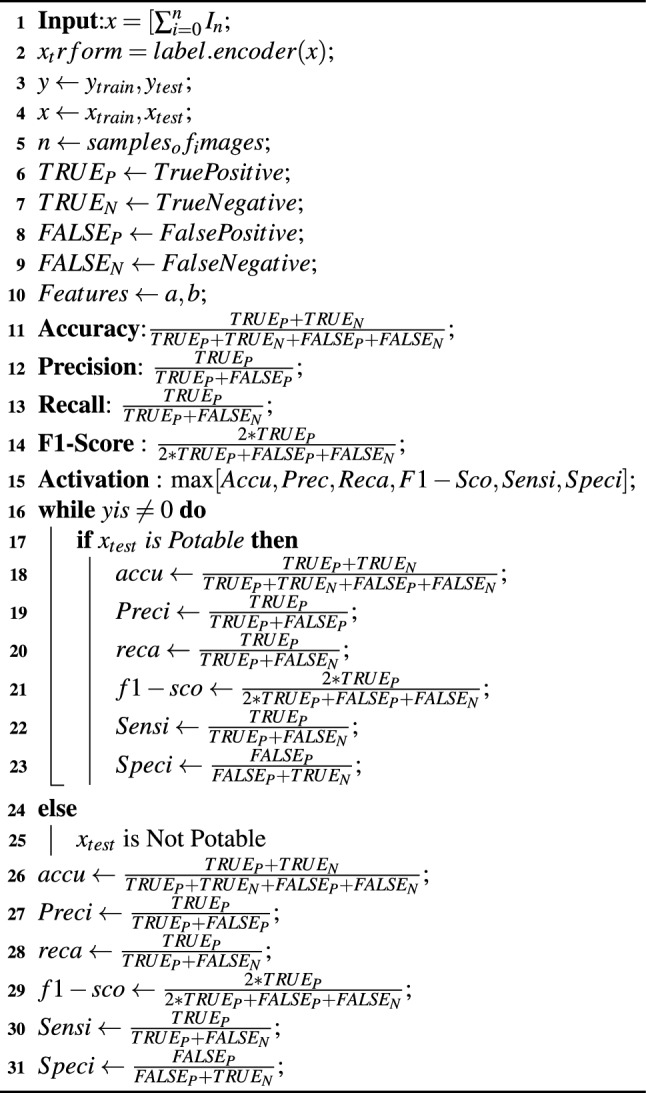
Algorithm for water quality classification

**Algorithm 2:**
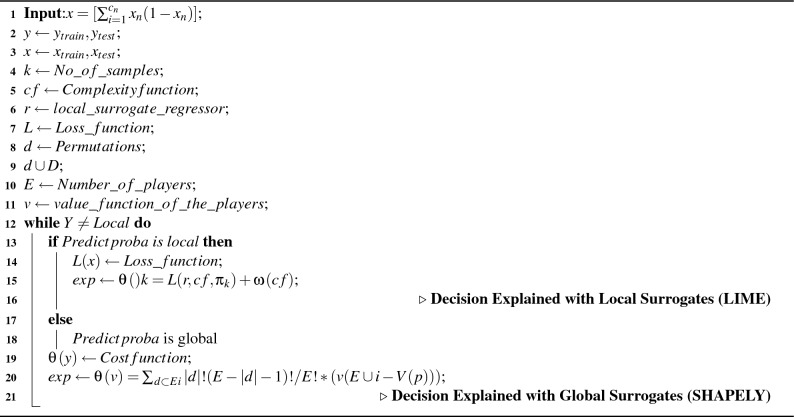
Algorithm for water quality Explanation

## Results

The water quality is assessed in the proposed work based on nine parameters such as pH value, Hardness (Total Dissolved Soils), Sulphate, Chloramines, Trihalomethanes, Conductivity, Organic carbon, and Turbidity. The target class for this dataset is Potability which is binary where 0 indicates that the water is not potable and 1 reflects its potability.

The dataset consisted of high missing values on sulphate and lower missing values on Chloramines and Trihalomethanes. The missing value imputation is hence performed and all the attributes are imputed for the missing values. The target class is converted into a numeric array for the processing of XAI models. This is done with the label encoder application of Python. The dataset is split with a ratio of 80:20 for training and testing.

**Figure 8:**
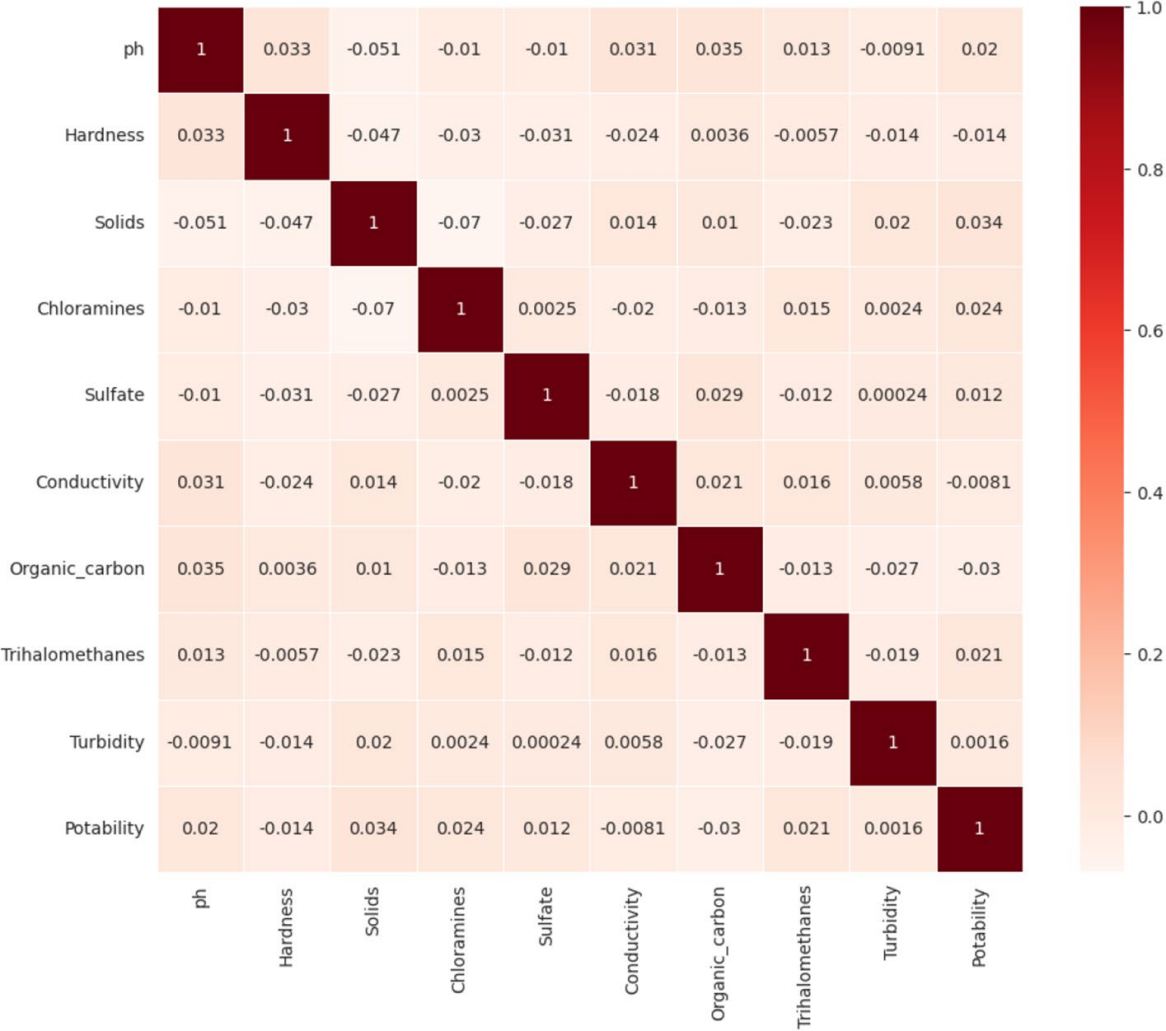
Correlation analysis for water quality attributes.

The correlation analysis is performed on the dataset. The attribute Hardness has a high correlation of 0.34 with the target attribute potability. The next best correlation value is 0.24, which is rendered by the attribute Chloramines, followed by 0.21 produced by the Trihalomethanes attribute. Turbidity is the next better parameter with a correlation value of 0.16. The correlation heat map between the attributes of interest and the target attribute is presented in Fig. 8.

The trained dataset is applied with SVM, LR, DT, RF and Gaussian Naive Bayes machine learning models. The SVM did not provide the desired classification and failed to converge for the portable data. The other models generated the results within the desired range and are presented in Table 6.

**Table 6:** Comparison of Metrics of Machine Learning Models on Water Quality.

Model	Accuracy	Precision	Recall	F1-Score
Logistic Regression	0.77	0.74	0.75	0.76
SVM	0.63	0.39	0.63	0.48
Gaussian Naive Bayes	0.996	0.994	0.992	0.997
Decision Tree	0.998	0.996	0.997	0.998
Random Forest	0.999	0.997	0.998	0.999

The sensitivity and specificity measurements for the Machine learning models are presented in Table 7. Considering the performance metrics, the results reveal the superiority of the RF model which generates a better outcome in comparison to the other models and thus it has been selected to be fed into the XAI model to provide enhanced interpretability, justifiability and transparency.

**Table 7:** Comparison of sensitivity and specificity for the machine learning models.

Model	Sensitivity	Specificity
Logistic Regression	0.76	0.75
SVM	0.55	0.54
Gaussian Naive Bayes	0.995	0.994
Decision Tree	0.996	0.997
Random Forest	0.998	0.999

**Figure 9:**
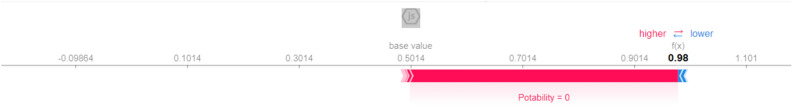
Force plot for water quality.

**Figure 10:**
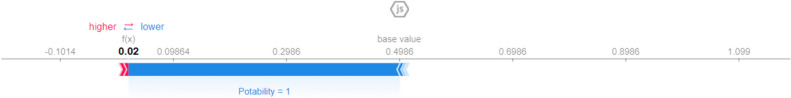
Force plot for potability.

The XAI model implementation is performed considering SHAPELY values in the pandas’ application. This application focuses on the value of each feature in determining the target attribute which is potability. The significance of every feature is assessed through the various applications of SHAPELY. The first XAI model generated is the force plot, which provides the minimum and maximum prediction score of the target attribute in a dataset. The blue colored contour shows that a low score is measured and the red color shows a high score. The values at the separation boundary have the highest priority attribute. The force plot is presented in the Figs. 9 and 10.

**Figure 11:**
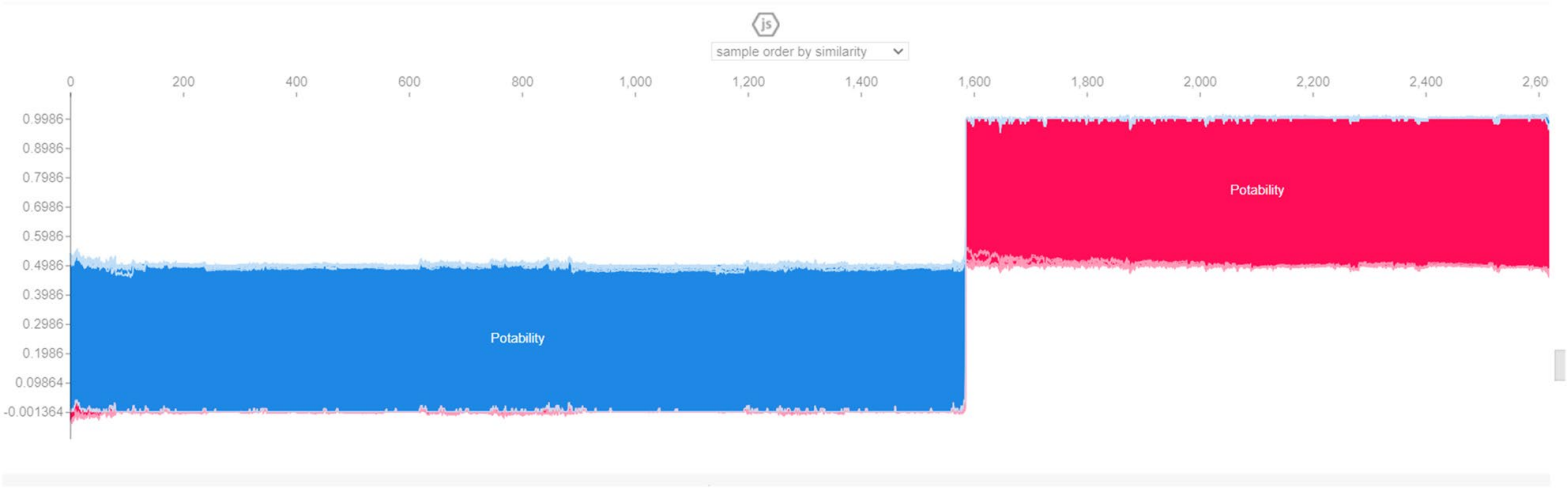
Test patch for potability.

The Global surrogate version of the force plot is presented in Fig. 11. The blue regions indicate no potability and the red-coloured regions indicate potability. The border areas of the intersection show the attributes which have higher significance for the feature selection. The Sulphate value of 444 at the point of intersection indicates its significance in explaining this test patch for the entire dataset.

**Figure 12:**
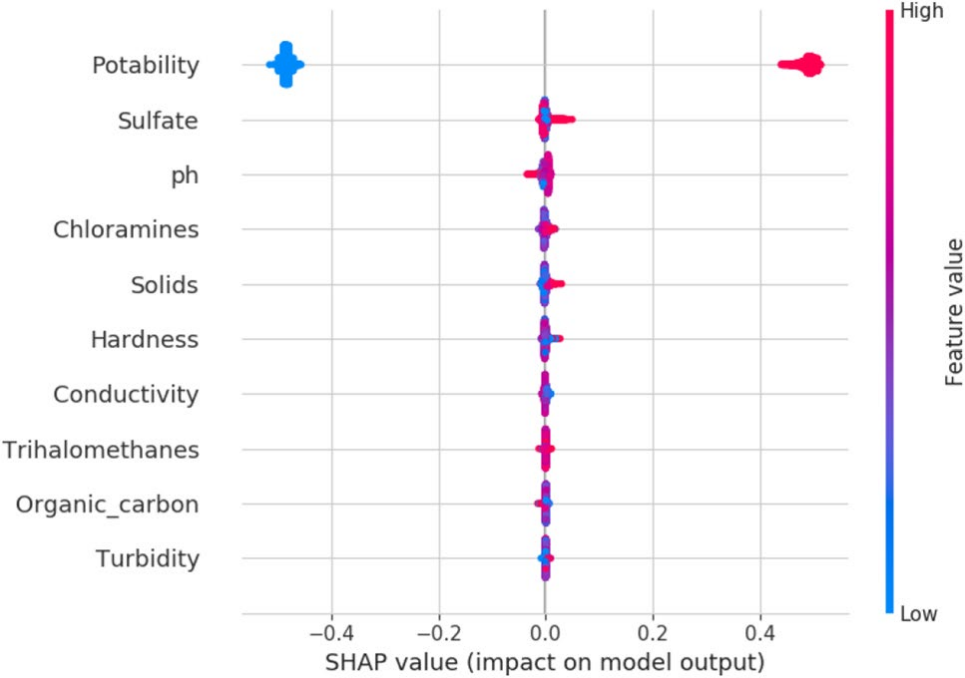
Summary plot for potability.

The next XAI application of SHAPELY is the summary plot. This plot describes the features in determining binary classification problems. This predicts the scale of low to high for two significant results. The blue contour indicates lower significance towards the prediction and red indicates higher significance. The summary plot is shown in Fig. 12. The Solids, pH, Sulfate, and Hardness show higher significance in determining the output.

**Figure 13:**
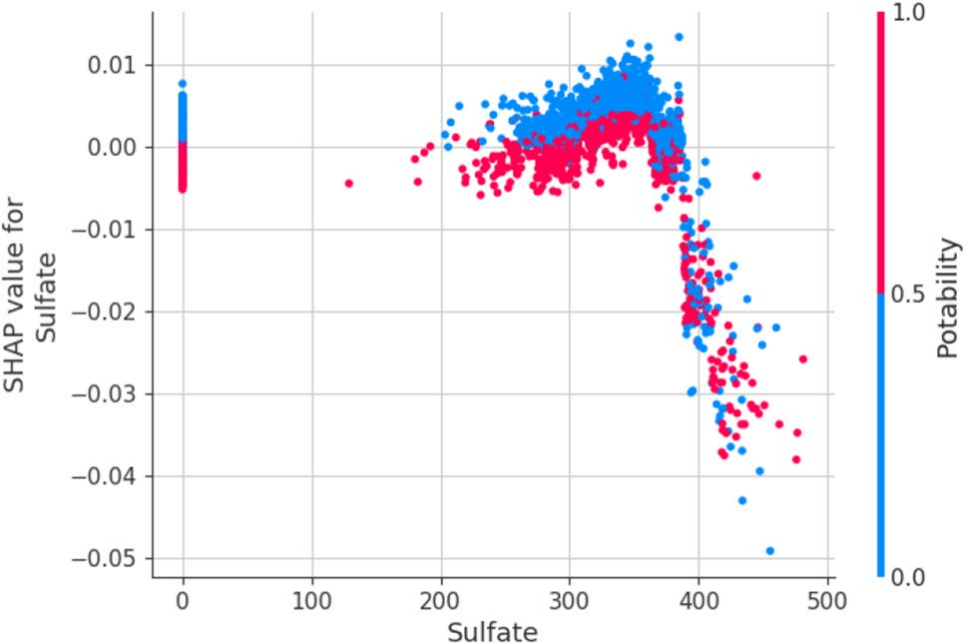
Dependency plot for potability.

The dependency plot shows the relationship between two features in the dataset. It provides the output in granular form with a variable-like result rather than simply a graph-like result of a Partial Dependency Plot(PDP). The relationship between the Sulphate and Potability is depicted in Fig. 13. The mid-range of the dataset provides more granular output, which shows that the Sulphate parameter values are more significant in determining the values of potability in the mid-range of the dataset.

**Figure 14:**
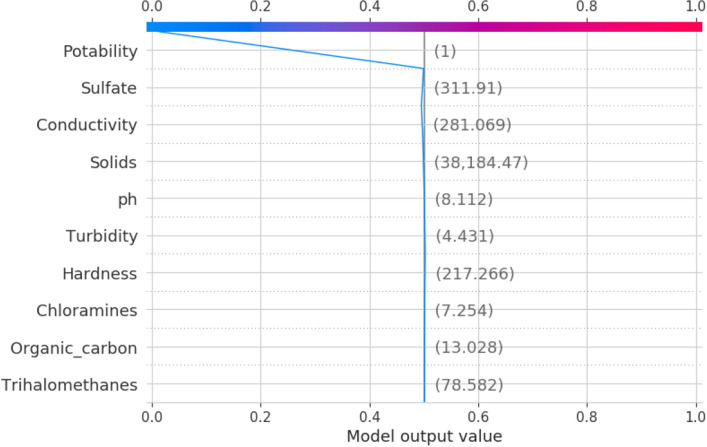
Decision plot for potability.

**Figure 15:**
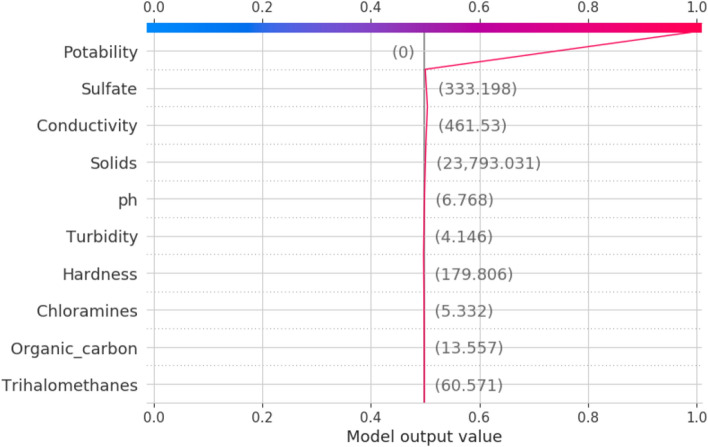
Decision plot for potability.

The decision plot, which displays how the values of the features affect the goal, is the final model of XAI. This plot is a local surrogate plot, which would only explain a certain data instance, in which what values of the attributes influence the decision to be 1 or 0 as the decision of the model. The decision plot for the potability as 1 is illustrated in Fig. 14. The potability 0 is illustrated in Fig. 15.

## Discussion

**Figure 16:**
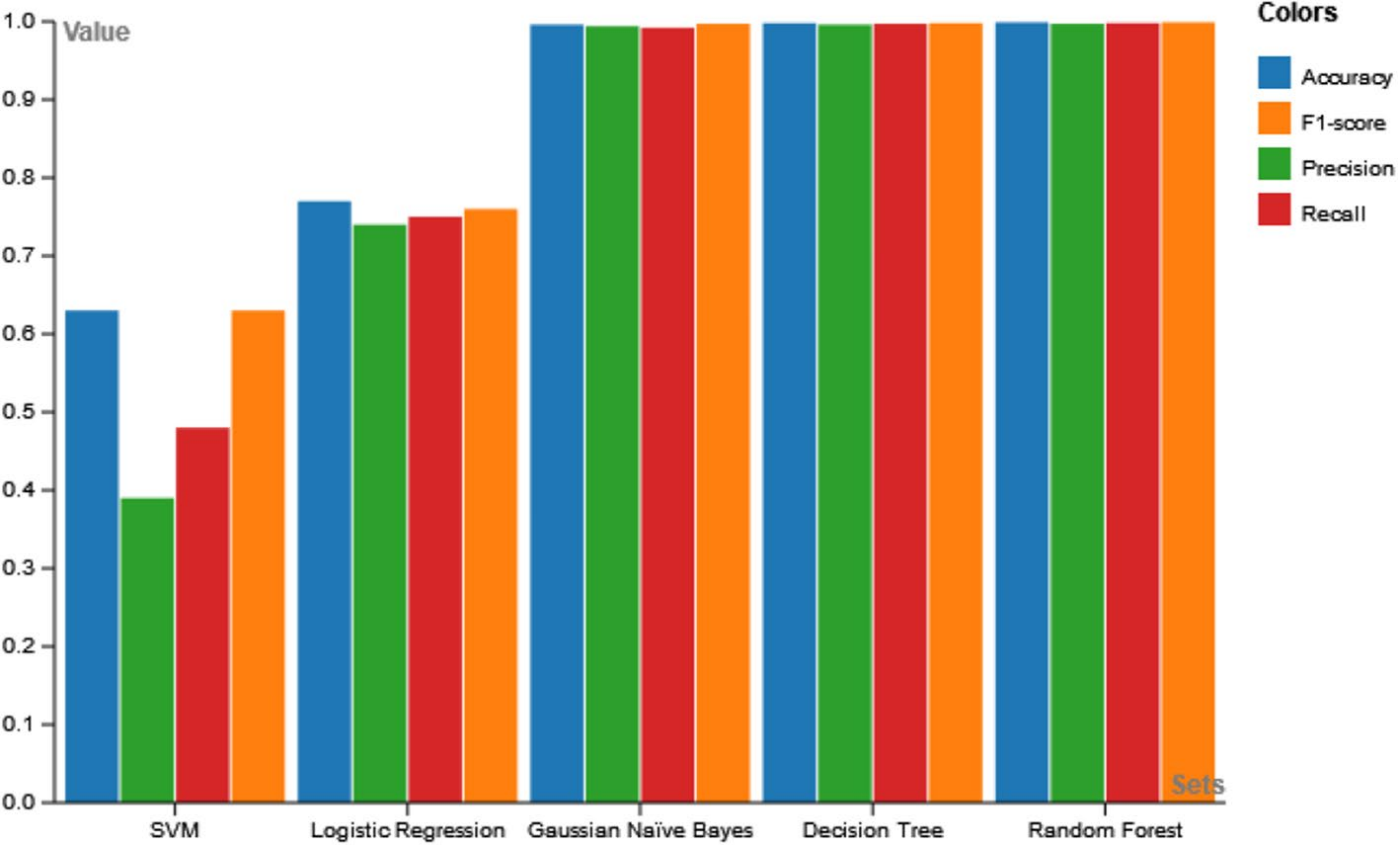
Comparative analysis of machine learning models used.

**Figure 17:**
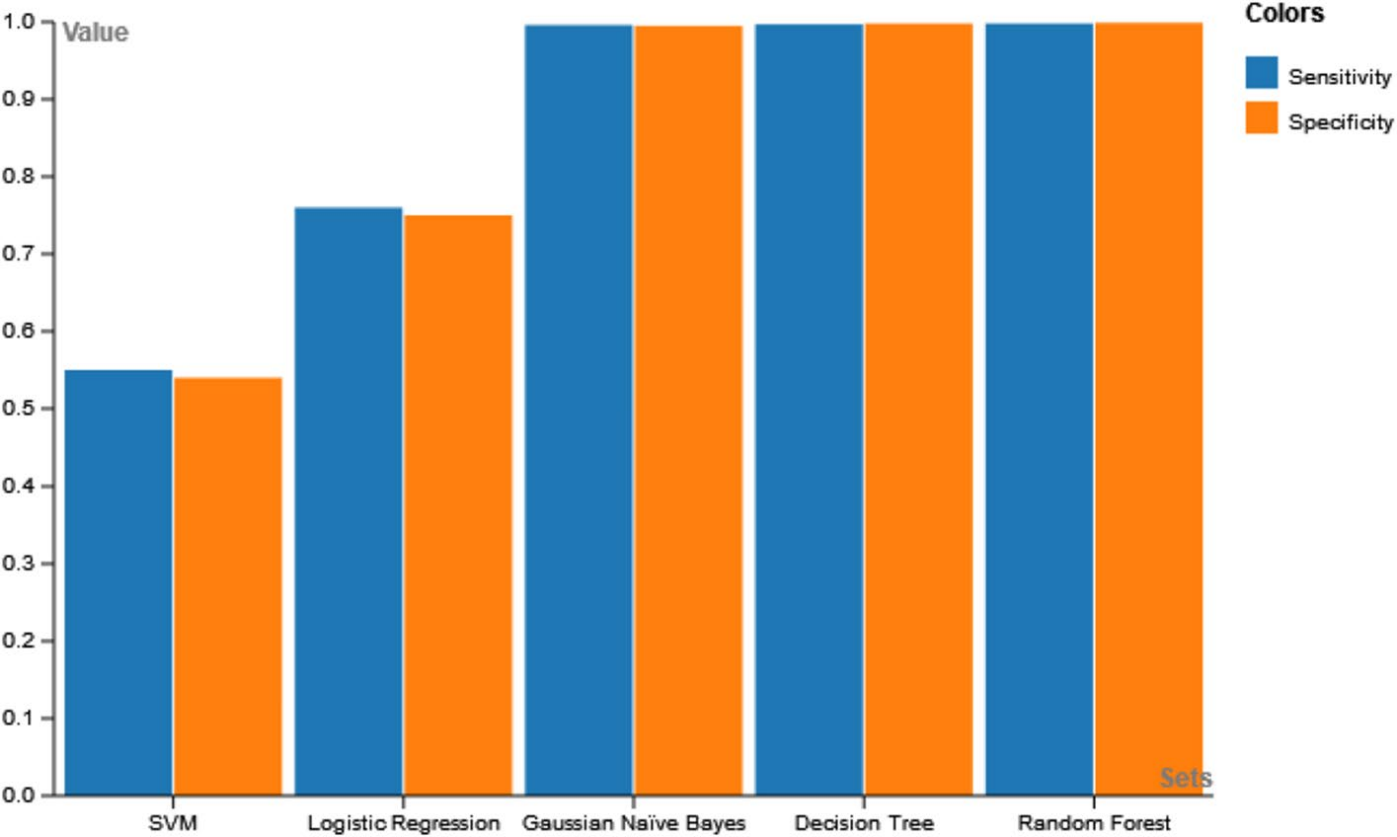
Comparative analysis of sensitivity and specificity.

The results of the experiment reveal the superiority of the RF model which generates an accuracy of 0.999 followed by DT, generating an accuracy of 0.998. The lowest accuracy is generated by the SVM model of 0.63. The RF is thus chosen for the implementation of the XAI model using SHAPELY. The comparative analysis of the aforementioned various models is depicted in Fig. 16, considering evaluation metrics accuracy, precision, recall, and f1-score. In the case of all the performance metrics, the RF model outperforms the other models. Figure 17 shows the comparison of the sensitivity and specificity measures. The RF model stands superior in these considerations as well. Thus, the discussion offers a visual representation and justification of the reasoning behind the choice of RF to be included in the XAI framework to offer explainability.

Apart from the selection of the RF model, SHAPELY provided five different models to explain the feature importance and relationships. The proposed work presented the force plot, summary plot, test patch, dependency plot, and decision plot. The Final decision plot explained how the classification is carried out using the corresponding values of the independent variables. Thus the black-box classification is explained in the white-box context of XAI. The following section describes the challenges and opportunities of the proposed work with an emphasis on future directions.

### Challenges

The proposed work may be influenced by the following challenges which are described in detail as follows,

#### Global unity

For the successful implementation of the system, a unanimously accepted implementation is essential. Unfortunately, water quality estimation and related research are limited to consideration of specific datasets acquired for a particular region, wherein the generated results may differ with the changes in geographic location. Thus the generated results can never be considered suitable on a global scale. The parameters that influence the water quality may also vary across the world, and hence the proposed work can never be considered as a universal solution.

### Training and re-training

The qualifying attributes that determine the quality of water vary across the globe and hence the proposed model needs to be re-trained^69^ when applied to a new environment of study. This would allow the model to unlearn and re-learn new environments. On the contrary, the complexity of the model would also increase. The accuracy and other performance metrics which are measured in the proposed work may drastically decrease as well in a different environment of study. Thus applying this model to versatile environments is complex and would be a challenging task.

### Subjective or quantitative

The trade-off from subjective analysis (which was done through fuzzy-based methods in the form of the Analytical Hierarchy Process (AHP) and The Technique for Order of Preference by Similarity to Ideal Solution (TOPSIS)) has improved the performance and ability to classify the models with better accuracy. However, the involvement of a subject matter expert is a missing point in the current research. Despite all the implementation and analysis from an engineering perspective, the involvement of an environmental scientist in any aspect of water research would contribute towards the enhancement of research quality.

### Confusing solids

The proposed work identifies Solids as the primary influencing factor that affects potability. In real-world applications, solids can be of any form. For example, in sewage water treatment plants it can be either mud, Fat-Oil-Grease(FOG), or any other substances. Every solid wastage has its way of filtration and impact on water quality, which makes the recordings unstable from time to time. The attributes of research are too complex to handle in real-life scenarios, which acts as an inevitable yet detrimental impact.

### Environmental challenges

Water resources are under serious threat due to water scarcity, water contamination, water conflicts and climate changes. Chemical and the municipal wastewater contaminates the water and endangering the life of the aquatic organisms and affect their ability to reproduce. This also makes them an easier prey to their predators. The food cycle and livelihood of the human is also greatly affected by the water contamination. Chemical substances make the water hard to recycle and consume by reducing the regeneration ratios.

### Water quality and industrial sustainability

The era of Industry 5.0 focuses on the consumer centric industrial evolution with the idea of environmental sustainability. The futuristic technologies evolve with the improvement of technical viability, with the mission of sustainable development in the environmental aspects. Since the water is an irreplaceable and finite, the demand of the water is increasing with the industrial evolution and the water requirements on manufacturing and production industries would be very much essential as ever. The challenge is enhancement of the water harvesting, recycling and conservation. For all the above said processes quality of the water is the common essential requirement. Thus the quality of the water is more critical in all futuristic technological developments.

### Research finding of the proposed work

The following items are presented as the findings are outcomes of the proposed work

- The proposed work performs an exploratory analysis with XAI implementation providing an ability to improve the reliability of machine learning models providing explanation and transparency to the classification process.
- The proposed work acquires data from a single dataset, where the performance of classification yields optimized results. This result may vary if the model is subjected to a different dataset constituting different features and instances.
- The XAI reveals the most significant features contributing towards classification results and also explains the same.
- The best fitting machine learning model is chosen for the explanation through an exhaustive analysis and evaluation of all the models considering the essential performance metrics. Thus the results produced by SHAPELY can be considered as the most reliable and acceptable. 
- The proposed work also suggests the importance of the subject matter expert, which can extend the usability of the proposed model at the universal level.
- The predictions of the proposed work with the support of an explainer, helps end users and consumers to understand the quality of the water they use.
- The features related to the classification and explanation, can be further controlled to diminish the levels of chemicals and pollutants in water recycling.
- Total dissolvable solids quantification and the feature weights for the same determine the levels of filtration and carbon purification required in the recycling plants.
- The proposed work brings insights of pollutants on the seashore and how the explainabilty can support the impurity estimations for such conditions also.

## Conclusion

Water quality management impacts almost all aspects of life on earth and clean water is a basic necessity. The proposed work is extremely relevant in this regard wherein an exploratory analysis conducted to analyze and control the factors that deteriorate the quality of the water. The impact of these factors is explained using XAI models. The contribution of the XAI model lies in its ability to explain the role of the underlying parameters towards the classification of water being potable or not, based on their relative importance and unique properties. The XAI model uses SHAPELY considering the probabilistic prediction generated from the Random Forest classifier. This RF model in this regard is chosen as it yields the highest accuracy of 0.999 with sensitivity and specificity of 0.999 and 0.998, which is found to be superior in comparison to the other state-of-the-art models considered in the study. This justifies the reason for the RF to be selected for XAI implementation. The proposed model identifies the parameter “solid” as the most significant in terms of its impact on the potability of water. The proposed model yields optimized and explainable results considering the dataset used in the study. Future work may involve more complex and heterogeneous datasets to generate predictions. In such scenarios, the metric evaluations may differ. The usage of deep learning algorithms could further enhance the examination the solid sediments and generate classification results based on their mass, dimensions, and shape. The use of XAI in such a model would ensure a better explanation of factors relevant to the solid sedimentation in water.

## Data Availability

The data that support the findings of this study are available from the corresponding author, upon reasonable request.
